# Across the Hall from Pioneers

**DOI:** 10.3390/v13030491

**Published:** 2021-03-16

**Authors:** Alan Rein

**Affiliations:** HIV Dynamics and Replication Program, National Cancer Institute, Frederick, MD 21702, USA; reina@mail.nih.gov

**Keywords:** retroviruses, virus maturation, viral proteins, HIV, post-translational modifications, translational suppression

## Abstract

I was fortunate to be associated with the lab of Stephen Oroszlan at the US National Cancer Institute from ~1982 until his conversion to Emeritus status in 1995. His lab made groundbreaking discoveries on retroviral proteins during that time, including many features that could not have been inferred or anticipated from straightforward sequence information. Building on the Oroszlan lab results, my colleagues and I demonstrated that the zinc fingers in nucleocapsid proteins play a crucial role in genomic RNA encapsidation; that the N-terminal myristylation of the Gag proteins of many retroviruses is important for their association with the plasma membrane before particle assembly is completed; and that gammaretroviruses initially synthesize their Env protein as an inactive precursor and then truncate the cytoplasmic tail of the transmembrane protein, activating Env fusogenicity, during virus maturation. We also elucidated several aspects of the mechanism of translational suppression in *pol* gene expression in gammaretroviruses; amazingly, this is a fundamentally different mechanism of suppression from that in most other retroviral genera.

Dr. Stephen Oroszlan joined the National Cancer Institute-Frederick Cancer Research Center in 1976 and maintained a research laboratory there until his conversion to Emeritus status in 1995. In this relatively brief time-span, he and his laboratory made many fundamental discoveries that helped to lay the foundation for contemporary retrovirology. I had the extraordinary good fortune to be located “across the hall” from the Oroszlan lab, and I heard about many of these discoveries while they were still in progress. In this paper I will briefly tell four stories of my own research on retroviruses; it will be clear that each of them is based on the findings of the Oroszlan lab.

Much of the basic work of the Oroszlan lab consisted of determining amino acid sequences of proteins obtained from retrovirus particles. While sequencing of DNA and RNA has now become so routine and so fast that sequences are practically taken for granted, this was not true when Steve and his lab were conducting this research (both Maxam–Gilbert and Sanger DNA-sequencing methods were only introduced around 1977). Moreover, retroviral proteins are, like those of many viruses, initially synthesized as polyproteins and then produced by proteolytic cleavage of these polyproteins. Therefore, the termini of these cleavage products cannot be inferred from a DNA sequence, which will only determine the amino-acid sequence of the polyprotein. Thus, one of the fundamental contributions of the Oroszlan lab was the painstaking work of purifying proteins from viruses and determining their N- and C-terminal sequences. (This “maturation cleavage” of the polyproteins is absolutely essential for virus replication. In most cases it is carried out by virus-coded proteases; these are among the crucial targets in the highly active antiviral therapy used today to control human immunodeficiency virus-1 (HIV-1) replication in infected people. Identification of the cleavage sites also reveals the target sites and preferences for these viral proteases; this information was invaluable in the development of protease inhibitors.) This careful analysis of the viral proteins also led to the identification of post-translational modifications and to examples of “recoding”, in which the amino acid sequence of the protein does not correspond exactly to the sequence predicted from the sequence of the template mRNA. I will describe examples of each of these phenomena in the sections below.

## 1. “Cysteine Arrays” in Nucleocapsid Proteins

The taxonomy of retroviruses uses the convention of the family Retroviridae, with two subfamilies of Orthoretrovirinae and Spumaretrovirinae. There are six genera (each with multiple species) within the Orthoretrovirinae, or Orthoretrovirus subfamily, most of which will be mentioned below. Spumaretrovirinae members differ in many important respects from orthoretroviruses and will not be considered further in this report. All orthoretroviruses contain a small, very basic protein called the “nucleocapsid” (NC) protein. This protein coats the RNA in the interior of the mature virion. Using the classical techniques of protein chemistry, Henderson et al. [1] determined the entire 56-residue sequence of the NC protein of two murine leukemia virus (MLV) isolates in 1981. In discussing their findings, they noted the presence of a specific arrangement of cysteines, glycines, and histidines, i.e., C-X_2_-C-X_3_-G-H-X_4_-C. This sequence motif, which they called a “cysteine array”, had previously been observed to occur twice within the NC protein of avian retroviruses (MLV and avian retroviruses are in different retroviral genera, namely, gammaretroviruses and alpharetroviruses respectively, and there is very little sequence conservation between them). In the next few years, sequences were obtained for many retroviral NC proteins, and it was striking to note that each of them contained one or two perfectly matching copies of this array (except for occasional substitutions of the glycine). Remarkably, the same sequence motif was also found in the *Drosophila* transposable element *copia* and in cauliflower mosaic virus, in which virions contain DNA but, as in retroviruses, sequences are copied between RNA and DNA in the replication cycle [2].

Jeremy Berg made an important contribution to the field by suggesting that the regularly spaced cysteine and histidine residues in these proteins might coordinate a metal ion [3]. This ion might be zinc, as in the “zinc-finger” motif discovered in another nucleic acid-binding protein by Miller et al. [4]. In any case, the absolute conservation and spacing in the arrays seemed to signal some significant function, and methods for site-directed mutagenesis had recently become available. Rob Gorelick, a postdoc working with Lou Henderson in the Oroszlan lab, came to work with me around 1986, with the intention of changing individual conserved residues in the cysteine array of MLV.

In a series of mutants, Rob replaced each of the cysteine codons in the NC of a replication-competent MLV clone with a serine codon. This merely changes a single sulfur atom to an oxygen atom in the protein, but it disrupts its putative ability to coordinate a zinc ion. He then transfected the mutant genomes into cultured hamster cells and characterized the mutant viruses produced by these cells [5]. The results were striking and illuminating: the mutant genomes directed the production of superficially normal mature virions, but these particles contained very little viral RNA, as tested by Northern analysis. (The absence of a band of genomic RNA in these Northern blots could conceivably reflect degradation of the RNA, rather than a failure to encapsidate it. We subsequently obtained strong evidence against this alternative hypothesis by reverse transcription polymerase chain reaction (PCR) [6]). These fundamental results demonstrated that the conserved cysteines play a critical role in the packaging of the viral genomic RNA during virus particle assembly. Moreover, quantitation of packaged viral RNA and of the infectivity of the mutant particles revealed that the deficit in infectivity could not be fully explained by the deficit in viral RNA content [5]. In turn, this shows that the conserved cysteines are required for other viral functions, as well as for viral RNA encapsidation.

The functions of these conserved residues in NC proteins have been extensively investigated in subsequent years. Rob was interested in making analogous mutations in HIV-1, and ignored my well-intentioned, if naïve, advice that making the same mutant in a different virus (HIV) was unnecessary because it would merely give the same answer as he had already obtained in MLV. In fact, changes in the conserved cysteines in the two arrays in HIV-1 NC were found to impair encapsidation of viral RNA [7,8], just as I had predicted; but on the other hand, these results also earned Rob the offer of a research position in the new AIDS Vaccine Program in Frederick.

It also became clear that the conserved cysteines and histidines do indeed coordinate a zinc ion within the virion [9,10], as suggested [3]. Folding around the zinc evidently imposes a very specific 3-dimensional shape upon the stretch between the first and last cysteine in each array, while in contrast, the remainder of the NC protein appears quite flexible [11,12,13]. Aromatic and other hydrophobic residues within these zinc fingers engage in stacking interactions with guanosines in nucleic acids; this seems to be an important component in the specific binding of NC proteins to RNAs [11,12], in addition to the non-specific, electrostatic binding of the highly basic protein to the negatively charged nucleic acids.

Adding significant complexity, the NC proteins of most retroviruses contain two zinc fingers; their functions are not redundant and how their functions are divided between them is not understood [14]. Interestingly, MLV mutants in which cysteines and histidines are exchanged, so that they still retain the four zinc-coordinating residues but are structurally different from wild-type, successfully package viral RNA but are still defective with respect to viral replication [15]. NC proteins are crucial cofactors in reverse transcription, largely because of their nucleic acid chaperone activity, and the zinc fingers are major contributors to this activity [16]. The same is true of the zinc fingers in the parent Gag protein which, thanks to its chaperone activity, anneals a cellular tRNA molecule to the viral RNA in the assembling virion, positioning the tRNA to prime DNA synthesis in the next round of infection [17].

The array with an absolutely invariant spacing of cysteines and histidines, discussed here, is found in all orthoretroviruses and also in retroelements found in plants, flies, and yeast. These elements are only very distantly related to each other, and so the common sequence motif was a very strong hint that it had some profound functional significance. As discussed here, this turned out to be correct: it functions in RNA encapsidation during particle assembly, and also during reverse transcription. In this case, the motif could have been noted by anyone comparing sequences of such disparate genomes, using either protein- or nucleic acid-sequencing methods. The Oroszlan lab deserves credit not only for determining the sequences of a wide variety of retrovirus NC proteins, but for the perspicacity to realize that this conserved motif deserved further study. I was blessed with the good fortune of proximity to the lab and specifically the enterprising spirit of Rob Gorelick, who initiated the mutagenesis studies described above and helped uncover the multiple functions of NC proteins in a series of investigations spanning many years.

## 2. N-Terminal Myristylation of Retroviral Gag Proteins

As indicated above, a major effort of the Oroszlan lab in the 1970s and 1980s was the determination of amino acid sequences in retroviral proteins, largely using classical methods of protein chemistry. These methods include Edman degradation, in which the amino acids are sequentially removed from the N-terminus and identified. It is based on a reaction with the unique free amino group at the N-terminus of a protein. However, this method gave no results for matrix (MA) proteins, which are the cleavage product derived from the N-terminus of the Gag precursor: evidently the N-termini of MA and of Gag do not have a free amino group, but are ”blocked” to Edman degradation (with a single exception noted below).

The chemical nature of the block was completely unknown until it was determined by Henderson, Krutzsch, and Oroszlan in 1983, studying the MA protein of MLV [18]. They digested proteins isolated from MLV particles, and further analysis by gas chromatography/mass spectrometry identified the group at the blocked N-terminus as myristic acid, a 14-carbon saturated fatty acid, attached to the N-terminal glycine by an amide linkage [18]. At the time, this protein modification had not been described before (although a report on myristylation of another protein, the catalytic subunit of cyclic AMP-dependent protein kinase [19], preceded the publication from the Oroszlan lab, to Steve’s great chagrin). Alan Schultz, who along with Lou Henderson was a senior scientist in the Oroszlan lab, quickly showed using metabolic labeling that Gag proteins from a number of retroviral genera were myristylated [20].

What was the functional significance of this new modification? In all of these cases, the initiator methionine codon in the gene was followed by a glycine codon, so it seemed reasonable that in all cases, the methionine was removed and the newly exposed N-terminal glycine was myristylated. Therefore, we decided to delete or replace the glycine codon in a retroviral *gag* gene. If we were correct that N-terminal glycine is required for myristylation, then the mutant should lack the modification but otherwise be almost identical to the wild-type protein. My lab-mate Nancy Rice had attended a course at Cold Spring Harbor in the summer of 1983, where Mark Zoller, a young colleague of Michael Smith, taught the nuts and bolts of site-directed mutagenesis. As I had a fully infectious molecular clone of Moloney MLV, I took advantage of the protocols Nancy had brought back to replace the glycine codon with an alanine codon, or to delete the codon entirely, in this clone. I must emphasize that this was before the days of kits! Every step in the mutagenesis procedure, which involved cloning the target region into the single-stranded DNA phage M13 and using the single-stranded sequence as template with the mutagenic oligonucleotide as primer, was laborious and uncertain of success.

We eventually succeeded in reconstructing the two full-length MLV genomes, one with the deletion and the other with the alanine substitution for the glycine codon at the 5′ end of *gag* [21]. When these mutant viral genomes were introduced into mouse or hamster cell cultures, the results were dramatic: the Gag protein was present in cell lysates, but, unlike the wild-type controls, it could not be metabolically labeled with [^3^H]-myristic acid. Most interesting, there was no detectable virus released from the cells, and no particles or recognizable virus-specific structures were seen in electron micrographs of cells expressing the mutants. Simple cell-fractionation experiments also showed that the mutant Gag protein was soluble in cell lysates, whereas in the controls, wild-type Gag protein was largely bound to membranes or other large, pelletable structures in the cell.

These results confirmed that the N-terminal glycine was necessary for myristylation, and also demonstrated that the myristyl modification was crucial for membrane association and virus production by the MLV Gag protein. We suggested [21] that membrane association is a way of concentrating the protein within the virus-producing cell, and this might be one way that it contributes to particle assembly. Alan Schultz and I followed up these original observations with a further analysis of the behavior of the unmyristylated mutant Gag protein. We found that it was metabolically stable within cells and did not undergo the maturation cleavages that occur after the release of wild-type virus [22]. We also reported that it did not participate in intracellular particle assembly, even in the presence of a normally assembling wild-type MLV Gag protein. It should be noted, however, that these experiments were all performed with stably transfected cells, in which the expression of the transfected genes is at a very low level; it seems possible that the limited expression of the mutant Gag contributed to its failure to assemble.

HIV-1 Gag is also, like MLV Gag, myristylated at an N-terminal glycine [23,24], and subsequent studies on HIV-1 have generally been quite congruent with our MLV results [25]. One difference is that unmyristylated mutant HIV-1 Gag protein does co-assemble with myristylated Gag [26], and in fact assembles on its own in the cytosol into virus-like particles that are easily recognizable in transmission electron micrographs [27]. However, these experiments used transient transfection, which gives a far higher expression level than stable transfection; as indicated above, it seems plausible that the difference in results is due to this difference in experimental protocol and is not a real difference between MLV and HIV-1. This possibility is also consistent with the fact that both MLV Gag and HIV-1 Gag proteins purified from *E.coli* will assemble into virus-like particles under the right conditions in vitro [28,29,30]; these proteins are not myristylated, since bacteria do not contain N-myristyl transferase.

Subsequent studies have shown that the Gag proteins of many, but not all retroviruses are myristylated at their N-termini. The exceptions include the non-primate lentiviruses, such as equine infectious anemia virus; this is surprising since they are so closely related to the lentivirus HIV-1. Another important exception is the alpharetroviruses such as Rous sarcoma virus (RSV); the N-terminus of their Gag protein is blocked by acetylation rather than myristylation [31]. As far as is known, only one retroviral Gag protein has an unmodified N-terminus: that of the lentivirus bovine immunodeficiency virus [32].

All of the retroviruses cited above assemble into particles at the plasma membrane. In contrast, in betaretroviruses such as Mason-Pfizer monkey virus (MPMV) (morphologically classified as a “D-type virus”), Gag is myristylated and initially assembles into an immature capsid near the centriole, within the cytoplasm. This pre-assembled immature capsid is then trafficked to the plasma membrane, where it associates with the Env glycoprotein and buds out of the cell. Interestingly, when the MPMV *gag* gene was mutated to prevent myristylation, it was found that intracellular assembly still occurred, but the mutant immature capsid did not migrate to the plasma membrane [33]. Thus, although the sequence in which assembly and membrane association occur is reversed in betaretroviruses relative to that in gamma- or lenti-viruses, the data in all of these viruses show that Gag myristylation is critical for its membrane association.

N-terminal myristylation has important implications not only for retroviral assembly, but also for malignant transformation. What we now call retroviruses were originally known as “RNA tumor viruses”, since the first retroviruses to be detected were oncogene-containing “acute transforming viruses”. The oncogenes in these viruses were, of course, captured from host cells. In many of these viruses, the oncogene in the viral genome is fused with the viral *gag* gene. In some cases, including Abelson leukemia virus, the Gag-Onc fusion protein is targeted to the plasma membrane by its Gag moiety, and this is crucial for its transforming activity; N-terminal myristylation is an essential component of this targeting [34,35,36].

The first viral oncogene to be identified was *v-src*, the transforming gene in Rous sarcoma virus (RSV). The transforming protein encoded by RSV, pp60^v-src^, does not contain retroviral Gag sequences (although it is not an intact copy of the parental cellular gene [37]). However, like the Gag and Gag-Onc fusions discussed above, it is myristylated at an N-terminal glycine, and the myristyl modification is essential for its plasma membrane localization and transforming activity [38,39,40].

Although myristylation is important in the targeting of most retroviral Gag proteins to the plasma membrane, it is not sufficient for their localization. In fact, it is now known that myristylated proteins are present in many cellular compartments. One other major element in the plasma membrane association of Gag proteins is the electrostatic interaction of positively charged residues on the surface of the MA domain with negatively charged headgroups in membrane lipids. This interaction is of prime importance in the case of alpharetroviral Gag proteins, which as noted above are not myristylated. It seems likely that a specific interaction between Gags and the phosphatidylinositol (4,5) bis-phosphate in plasma membranes is crucial in the specific association of Gags with the plasma membrane, rather than cytoplasmic membranes. A more fine-grained proposal is that the 14-carbon myristate chain at the N-terminus of Gag is inserted into the lipid bilayer, while a fatty acid chain from the membrane is inserted into a pocket in the protein. For a more complete discussion of these complex questions, the reader is directed to a thoughtful review by Dick and Vogt [41].

Very recently, it was discovered that a cellular protein, heme oxygenase 2, binds to the myristate chain on Gag proteins and reduces the amount of virus assembled from myristylated Gag protein [42]. This protein also binds a protein, Toll-like receptor adaptor molecule or TRAM, which modulates cellular inflammatory responses. The biological implications of these unexpected findings are unknown, but at least they imply that there is more to be learned about the significance of myristylation.

## 3. C-Terminal Truncation of Murine Leukemia Virus (MLV) Transmembrane Env Protein

All retrovirus particles contain the virus-coded Env protein. This protein is synthesized as a large precursor. Unlike Gag, it begins with a signal peptide, is translated in the rough endoplasmic reticulum, and trafficked through the secretory pathway to the cell surface. It is cleaved by the cellular protease, furin, into a large N-terminal fragment and a smaller C-terminal fragment. The N-terminal fragment, named surface glycoprotein (SU), is always glycosylated. The C-terminal fragment spans the plasma membrane in the virus-producing cell (and also the lipid bilayer at the surface of the virion), and is called the transmembrane protein (TM). Thus TM contains a stretch on the external side of the plasma membrane or viral membrane, called the ectodomain; a stretch of hydrophobic residues spanning the membrane; and a stretch on the cytoplasmic side of the plasma membrane or the inside of the virus particle, called the cytoplasmic tail. After cleavage of the precursor, SU and TM are held together in a complex, frequently by disulfide bonds, and three of these SU-TM heterodimers are associated into trimers on the virion. In the most general terms, when the virus infects a new host cell, the SU protein on the surface of the virion engages with a receptor on the surface of the cell; this interaction then leads to large conformational changes in the Env complex; the end result of these changes is that TM catalyzes the fusion of the viral and cellular membranes, so that the contents of the virion are deposited in the cytoplasm of the new host cell, where they will initiate the next cycle of infection.

The pathways by which the Env polyprotein precursors were processed into the SU and TM proteins in mature retrovirus particles were gradually worked out, using metabolic labeling and peptide mapping, initially largely by the lab of Ralph Arlinghaus. The murine leukemia viruses (prototype members of the gammaretrovirus genus) were the first for which this maturation pathway was thoroughly characterized. A major milestone in the analysis of gammaretrovirus biology was the publication, in 1981, of the complete DNA sequence of the Moloney MLV viral genome [43]; this made it possible to trace any experimentally determined protein sequence to its origin in the genome.

Unlike what is seen in most other genera, maturation of gammaretroviral Env proteins includes an additional cleavage: not only is the precursor cleaved into SU and TM, but the C-terminal 16 residues (roughly half of the cytoplasmic tail) are removed from TM. This was originally discovered using an approach which seemed like science fiction at the time, although it is now completely routine: beginning with the newly available sequence of MLV RNA [43], Green et al. synthesized a peptide representing the extreme C-terminus of the *env* gene, and raised a rabbit antiserum to the peptide. They found that this antiserum reacted with the intracellular Env precursors, but not with the TM protein in virions [44]. Interestingly, several lines of evidence suggested that this cleavage event, unlike the cleavage of the Env precursor into SU and TM, is catalyzed by the viral protease, and, like the cleavage of the Gag and Gag-Pol precursors, is a step in MLV maturation. For example, it does not occur in cells expressing Env but not Gag and Gag-Pol [45]. The Oroszlan lab later performed a careful census of the viral proteins in MLV particles, and showed that the 16-residue cleavage product is quantitatively retained within the mature virion (that is, it is in a 1:1 molar ratio to TM) [46], suggesting that it is not removed from the precursor before the particle has been fully formed.

The nomenclature regarding these MLV TM protein derivatives can be very confusing. The final TM protein has been called p15E (it is necessary to specify “E” for “envelope” to distinguish it from the Gag protein MA, which is called p15; the numbers here reflect the apparent molecular weight of the protein as estimated from its mobility in sodium dodecyl sulfate polyacrylamide gel electrophoresis (SDS-PAGE)). In this naming system, the TM precursor, still containing the 16 residues at its C-terminus, is called Pre15E, while the 16-residue cleavage product is called p2E. In some other papers, the TM precursor was called p15E and the mature form was p12E. However, an alternative terminology was introduced by Richard Lerner’s lab and is now generally accepted. They dubbed the 16-residue peptide the “R peptide” (explaining that this is because it is encoded at the “right” end of the genome, if 5′ to 3′ is, like English text, read from left to right [47]. However, some have suggested that “R” might really stand for Richard. In the long run, we may never know.)

In any event, it is evident that MLV encodes a TM protein that is 16 residues longer at its C-terminus than the TM protein which is present in the mature, infectious virion. This was quite unexpected, and raised the obvious question Why? Why does it take this complicated pathway, rather than simply encoding the mature TM protein? What is the functional significance of those 16 amino acids?

To gain insight into the functional significance of the R peptide and its removal during virus maturation, we made two mutants at the cleavage site [48]. In one, we introduced a stop codon, so that the viral genome encoded the mature, truncated form of the TM protein, rather than the wild-type extended form. (We originally called this mutant “p2E-“ but will now refer to it as “R-“.) In the other, we replaced the hydrophobic amino acid, leucine, at the N-terminal side of the cleavage site with the strongly polar amino acid arginine, anticipating that this drastic change in the sequence would prevent cleavage.

Very briefly, we found that both of the mutant TM proteins were incorporated into virus particles. The substitution of arginine for leucine blocked the cleavage, as expected. The deletion of the R peptide reduced the efficiency with which the virions infect new cells by about 10-fold, while we estimated that the mutation blocking the cleavage reduced infectivity approximately 100-fold. As R is evidently normally removed from the TM protein during virus maturation, we believe that the mature, truncated protein is never present in virus-producing cells. Thus it was of great interest to express R- Env in cells and look for any biological activity of the protein under these unnatural conditions.

**Figure 1:**
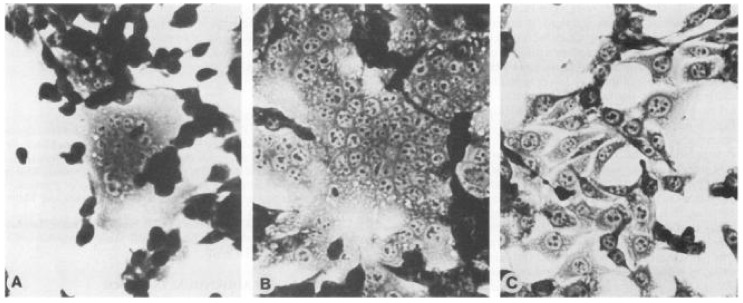
Syncytium Formation Induced by Expression of R- MLV Env. HEK 293T cells were transfected with a plasmid directing the expression of R- Env (**A**,**B**) or wild-type Env (**C**). Forty-eight hours later on’mouse cells were added to the cultures. The cultures were fixed and stained 3½ (**A**) or 24 (**B**,**C**) after addition of mouse cells (from ref. [48]).

In fact the results of these experiments were dramatic and illuminating. We found that when cells expressing R- Env (but not wild-type Env) were co-cultivated with cells displaying receptors for the virus, massive, rapid cell-cell fusion occurred (see Figure 1). In other words, the R- Env protein, which is the form present in mature, infectious virions, is highly active in inducing membrane fusion when it encounters the receptor on another membrane, while the full-length, immature form of the protein is not. This membrane fusion is, of course, the normal function of Env. The results suggest that the R peptide suppresses this fusogenic activity until the virus has been released from the virus-producing cell and undergoes maturation. Similar results were also reported at around the same time by Ragheb and Anderson [49].

A simple extension of these experiments revealed another intriguing property of MLV Env proteins [50,51]. As noted above, they are in trimers on the cell and viral surfaces. MLV Envs are polymorphic: that is, the Env proteins of different MLV isolates enter cells via distinct cell surface receptors [52,53]. We expressed two different Env proteins in the same cells [50]: one was R- (that is, fusogenic) with its SU directed towards one receptor, and the other R+, with an SU specific for a different receptor. We showed, by co-immunoprecipitation, that the two Env proteins were physically associated with each other. We then co-cultivated these cells with target cells displaying only the receptor for the full-length Env, not the R- Env, and observed a high level of cell-cell fusion. This fusion must result from the activity of the R- Env; in essence, this is complementation between the two Envs, with one binding a receptor on the target cell and the other inducing membrane fusion. The results suggest that in a mixed Env trimer, contact of the SU domain of one monomer with its receptor triggers the fusogenic action of the TM domain of another monomer.

How the R peptide suppresses the fusogenicity of MLV TM proteins is not understood. It is crucial to recognize that the machinery in TM for catalyzing membrane fusion is on the exterior side of the membrane, but it is sensitive to the effects of the R peptide, which is on the interior side. Fusion is initiated upon contact between the SU glycoprotein, located on the exterior side, and its receptor on a target membrane. In MLV, SU and TM are linked together by a disulfide bond [54], and the contact between SU and the receptor leads to rearrangement of the disulfides and loss of this covalent linkage between SU and TM [55]. One important clue emerged from the cryo-electron microscopic studies of Henrik Garoff and his colleagues [56]. They isolated Env trimers from MLV virions by solubilization with a non-ionic detergent, and noted a striking structural difference between the full-length and R- Env complexes: in the full-length complex, the C-terminal ends are closely associated with each other, but in the R- complex, the ends are splayed out. These results suggest that the R peptide holds the cytoplasmic tails together in the trimers and prevents the conformational change necessary for initiating fusion.

The roles of the different TM domains in membrane fusion have recently been further investigated by Marc Johnson and his colleagues, using detailed genetic mapping techniques [57,58]. Based on their results, they suggest that TM trimers contain coiled coils in their ectodomains and in their cytoplasmic tails and that these trimeric interfaces, which are on opposite sides of the membrane, must be aligned for successful TM function.

The power of the R peptide in suppressing the fusogenicity of a viral envelope protein was dramatically illustrated by appending it to HA2, the fusion protein of influenza virus [59]. This protein differs from that of MLV in that low pH is the trigger for fusogenicity. Despite this stark difference, the MLV R peptide inhibits fusion by HA. It would be very interesting to determine the effects of this addition on the 3-dimensional structure of HA protein, to see whether it tightens the trimeric association of cytoplasmic tails in this case as well as in MLV [56].

The presence of an inhibitory sequence at the C-terminus of an immature fusion protein is not confined to gammaretroviruses such as MLV. Mason-Pfizer monkey virus, a betaretrovirus, appears to have a very analogous system [60]. The viral protease appears to cleave the cytoplasmic tail in equine infectious anemia virus, enhancing viral infectivity [61]. In addition, there are many reports in the literature suggesting that fusogenicity of the Env proteins of lentiviruses, including HIV and simian immunodeficiency viruses, is inhibited by their long cytoplasmic tails (e.g., see [62,63,64]; the same may be true of the deltaretrovirus human T-cell leukemia virus [65]). In general, however, the biological significance of these observations is not clear.

One study concluded that HIV-1 could, under specific circumstances, evolve a system with striking analogy to the R peptide regulation discussed above in MLV. The HIV lipid bilayer is highly enriched in cholesterol. Waheed et al. [66] propagated HIV-1 in cultured cells in the presence of amphotericin B methyl ester (AME), a compound that binds cholesterol, and observed that AME inhibited virus replication. They were then able to select AME-resistant viral mutants. When these mutants were analyzed, they were found to have undergone sequence changes in the cytoplasmic tail of TM, and that these changes enabled the viral protease to cleave the tail, similar to the removal of the R peptide in MLV maturation. Experimentally induced truncations of the tail also led to AME resistance.

In conclusion, the R peptide seems to act as a “safety catch”, preventing activation of Env fusogenicity before the virus is released. We do not know why natural selection has led to this regulatory system in MLV. One possibility is that it is beneficial for the virus to prevent membrane fusion events, which would entail irreversible triggering of the Env fusion machinery, within the virus-producing cell; the suppression of activity in the cell would thus preserve the functional capabilities of Env for use in infection of a new host cell. Alternatively, the presence of a fusogenic Env protein within the virus-producing cell, with the possibility of encountering a receptor at an intracellular membrane, might be highly toxic to the cell; in this case, the benefit to the virus might lie in the protection of the virus-producing cell from this Env-induced damage. It must be remembered that MLV is, as far as we can detect, quite harmless to the cell, and when cultures of permissive cells are infected, they will continue to grow well and produce MLV indefinitely. These two hypothetical explanations for the selective advantage of the R peptide safety catch are, of course, not mutually exclusive.

## 4. Readthrough Suppression at the End of the MLV *gag* Gene

All orthoretroviruses encode Gag, the building block of the virus particle, and a far smaller amount of Gag-Pol, a fusion protein in which the viral enzymes are attached to (or near) the C-terminal end of Gag. It seems likely that synthesizing the enzymes fused to Gag is a way of ensuring their encapsidation into nascent virions, since the Gag moiety of the Gag-Pol fusion protein can presumably engage in homotypic interactions with the Gag proteins during virus assembly. Since the enzymes (reverse transcriptase and integrase, and—except in alpharetroviruses—protease) are only used catalytically, it is not surprising that they are produced at much lower levels (on the order of 1/20) than Gag. In fact, many experiments have shown that the ratio of Gag-Pol to Gag is critical for viral replication: both too much and too little Gag-Pol is highly detrimental.

How the viral genome directs the cell to synthesize large amounts of Gag and small amounts of Gag-Pol is an interesting problem with unexpected answers. The coding sequences for the enzymes are immediately 3′ of the *gag* gene in the viral genomes. Many virologists around 1980 tended to assume that the cell would splice a small fraction of the viral transcripts so as to remove the stop codon at the end of *gag*, allowing a few ribosomes to continue translation beyond *gag* and into *pol*. However, the attempts to find the hypothetical spliced RNA were uniformly unsuccessful, eventually forcing investigators to search for other possibilities.

Based on careful analysis of pulse-chase data of MLV-infected cells using monospecific antisera, Jamjoom et al. [67] pointed out that both Gag and reverse transcriptase (RT) seemed to be formed from a very large common precursor, but that Gag is synthesized in much larger quantities than RT. To explain these observations, they suggested that there might be some kind of translational control limiting translation of RT. Another fascinating early clue was reported in 1978 by Philipson et al. [68]. They purified the genomic RNA from MLV virions and added it to rabbit reticulocyte extracts for in vitro translation. They found that under these conditions, the RNA encoded primarily Gag and a much smaller amount (estimated at 1/50) of a Gag-Pol fusion protein, just as Jamjoom et al. had seen in infected cells [67]. However, if the in vitro translation system was supplemented with a purified amber (i.e., UAG) suppressor tRNA, the amount of Gag decreased and the amount of Gag-Pol increased. These findings led them to propose that the *gag* and *pol* genes are separated by a UAG in the viral RNA and that for some reason, termination at this UAG termination codon is normally somewhat inefficient. In fact, when the sequence was published a few years later, the prediction that the two genes are separated by a single UAG codon was confirmed [43]. Translation of a UAG codon at a frequency of 2–10% of that of a sense codon, while inefficient compared to the sense codons, is estimated to be ~1000 times the normal readthrough of UAG [69].

The actual mechanism of MLV *pol* expression was clarified by a single discovery in the Oroszlan lab a few years later [70]. Yoshinaka and Luftig had previously detected protease activity in MLV particles [71] and had accumulated evidence that this protease was responsible for the maturation of the virions. Yoshinaka came to work in the Oroszlan lab and, over a period of several years, performed the Herculean task of purifying the minuscule amount of protease from MLV particles in quantities sufficient for direct biochemical analysis. He found that the N-terminal sequence of the viral protease was T L D D Q G G Q… Comparison of this amino-acid sequence with the published sequence of viral RNA [43] immediately revealed the mechanism of synthesis of protease (the N-terminal portion of “Pol”, the precursor of the virus-coded enzymes). The last four codons of the *gag* open reading frame are T L D D. They are followed by the UAG termination codon separating *gag* from *pol*, and then the *pol* sequence, beginning with G G Q. This result showed that the Gag-Pol fusion protein is made by the inefficient translation of UAG as glutamine, and is then cleaved before the threonine, four residues N-terminal to the inserted glutamine. In fact, as the Oroszlan lab had already shown, the great majority of NC protein (the C-terminal cleavage product of the Gag polyprotein precursor) is also lacking the TLDD, the last four residues encoded by *gag* [46]; in other words, the viral protease normally cuts both the Gag and Gag-Pol proteins on the N-terminal side of the threonine.

I spent the next few years studying the mechanism of MLV *pol* expression in a close collaboration with Judith Levin of the National Institute of Child Health and Human Development, together with Ya-Xiong Feng, an extremely talented, knowledgeable scientist who started in Dr. Levin’s lab and later, because of the complexities of her visa situation, moved to my lab. We were also joined, in the early stages of this work, by Dolph Hatfield, a tRNA expert in the National Cancer Institute.

Our first experiments addressed the identity of the tRNA responsible for insertion of glutamine in response to the UAG codon. We were very surprised to read a paper by Yoshiyuki Kuchino, a prominent member of the tRNA community, reporting that the abundance of a minor species of glutamine tRNA, possessing activity capable of translating a UAG within the tobacco mosaic virus genome, is greatly increased by MLV infection [72]. Ya-Xiong and Dolph tried to follow up on this report. They programmed a reticulocyte lysate in vitro translation system with transcripts of cloned MLV DNA and showed that the translation past the end of *gag* in this system was dependent upon added tRNA [73]. However, they found the same level of this translational-suppression activity in extracts from control as from MLV-infected mouse cells. Moreover, the chromatographic profiles of glutamine acceptor activity from infected and control cells were indistinguishable. Thus, their conclusions differed from those of Kuchino et al. [72]: the data obtained by our group [73] indicate that the UAG at the end of MLV *gag* is translated as glutamine by a normal cellular tRNA.

It was of interest to determine whether normal mammalian cells also contain tRNAs capable of translating UAA and UGA in the context of the end of MLV *gag*. Therefore, we replaced the UAG with UGA or UAA, and tested for suppression of these termination codons, both in vitro and in mammalian cells [74]. We found that MLV genomes containing either of these codons in place of the wild-type UAG were fully replication-competent, with no obvious quantitative or qualitative phenotypic differences from wild-type MLV. Thus, the hamster cells and mouse cells we used in these experiments contained tRNAs which could translate UGA and UAA, as well as UAG. This was particularly noteworthy in the case of UAA, since suppression of this termination codon had not previously been described in cells of higher eukaryotes. When transcripts from these mutant genomes were tested in reticulocyte lysates, we found that UGA was translated as efficiently as UAG, but UAA was not; however, supplementation of the lysates with additional tRNA from mouse cells or rabbit liver led to translation of UAA at the same level as UAG or UAA.

These results [74] show that mammalian cells contain tRNAs which can insert amino acids in response to UAA or UGA, as well as UAG, in the context of the MLV *gag-pol* junction. What is the normal function of these tRNAs? Assuming that they participate in “normal” cellular protein synthesis, what amino acids do they insert? As an approach to this question, we performed experiments to identify the amino acids inserted in response to these termination codons in the MLV context [75]. These experiments were completely dependent upon the expertise of the Oroszlan lab, particularly that of Terry Copeland. We designed a very small construct for in vitro transcription, with only 7 codons on the 5′ side of the termination codon. The construct included 2 MLV codons 5′ of and 19 codons 3′ of the termination codon; this turned out to be sufficient viral sequence to induce suppression. We then transcribed this construct, translated the RNAs in reticulocyte lysates in which one amino acid was radioactive, and then analyzed the translation product by Edman degradation and measurement of the radioactivity released after each step. These measurements showed that UAA, like the wild-type UAG, is translated as glutamine; in contrast, UGA could be translated as cysteine, tryptophan, or arginine.

How can we explain the choice of amino acids inserted at these termination codons? In fact, each of them is encoded by a triplet differing from the termination codon at only one base: glutamine is encoded by CAA and CAG, and perhaps it is not surprising that tRNAs with anticodons pairing with these triplets could also pair with UAA and UAG, exploiting “first-position wobble” pairing. We have no information on whether UAA and UAG are translated by the same or different glutamine tRNAs. Of the three amino acids inserted in response to UGA, tryptophan is normally encoded by UGG, cysteine by UGU and UGC, and arginine by CGA, all differing at only one position from UGA. It is also fascinating to note that there is precedent for suppression in mammalian systems of UAG as leucine [76] and UGA as serine [77], but these are not observed in the MLV context. This raises the possibility that the context not only dictates translation of a termination codon, but also the identity of the tRNA that will participate in the translation. It seems possible that this is partially responsible for the discrepancy between the results of our group [73] and those of Kuchino [72]: the assay used in the latter study for detecting glutamine suppressor tRNA relied on translation of a UAG in tobacco mosaic virus RNA, not MLV RNA.

We also used the rabbit reticulocyte in vitro translation system to investigate the “context” in the MLV RNA responsible for inducing readthrough suppression [78]. Immediately 3′ of the UAG, there is an 8-base purine-rich sequence; this is followed by a sequence that can be folded into a stem-loop. This loop would contain 6 C residues. Nineteen residues beyond this stem is a run of 6 G’s. It thus seems possible that these bases form a pseudoknot, in which the bases in the loop of a stem-loop are paired with bases distal to the loop [79]. We used a construct containing the 57 bases that are 3′ to the termination codon in MLV, as we had found [75] that these were sufficient for suppression. We found [78] that each single-base change we made in the 8-base sequence prevented readthrough, but changes to the sequence that preserved the potential base-pairing in the putative pseudoknot were permitted. Thus, the results indicated that the signal for suppression involved both the 8-base sequence and the pseudoknot. Parallel analyses by Felsenstein and Goff [80] and Wills et al. [69] reached generally similar conclusions about the nature of the context promoting suppression.

One study of the structure of the MLV pseudoknot came to particularly interesting conclusions [81]. Houck-Loomis et al. performed nuclear magnetic resonance (NMR) on the pseudoknot over a range of pH values. Their data indicate that the RNA is in equilibrium between two different conformations, one of which leads to very efficient UAG translation, while the other does not. In other words, the efficiency of readthrough, according to this proposal, corresponds to the fraction of the molecules that are in the active conformation. Further, they showed that the pH of the buffer in in vitro translation has a very strong effect on readthrough frequency, and that this effect agrees with their model of the two structures. They identified a specific adenosine residue which is protonated at the lower pH, leading to the shift in conformation of the pseudoknot. They also tested specific mutants, and again found perfect agreement between the effects on the structure and the effects on translational suppression in vitro. Their work suggests that translation of the UAG is not inefficient, as we might have assumed; rather, they propose that it is highly efficient on those mRNA molecules that are in the active conformation. Further tests of this hypothesis are eagerly anticipated.

Inspection of the (now widely available) DNA sequences shows that there is a fundamental difference between *pol* expression in different retroviral genera. In gammaretroviruses (see above) and epsilonretroviruses, the *gag* and *pol* genes are in the same reading frame, separated by a single termination codon. However, in other orthoretroviruses, the *pol* gene is in the (-1) frame relative to *gag,* and the *gag* and *pol* reading frames overlap. In these viral genera, Gag-Pol is again expressed by means of highly unusual behavior of the translational apparatus. At a specific point within the overlap region, a fraction of the translating ribosomes inserts an amino acid, but only advances two, rather than three bases. This “ribosomal frameshift” puts this subset of the ribosomes into the *pol* (-1) frame, and from there they proceed to translate Pol. This mechanism was first described in the alpharetrovirus Rous sarcoma virus by Jacks and Varmus [82], and they subsequently characterized it in some detail in the lentivirus HIV-1 [83]. In some genera, there are two successive frameshifts, rather than one [84]: these frameshifts are more efficient than those in single-frameshift viruses such as alpharetroviruses and lentiviruses. The contexts dictating the frameshift have been studied in detail. While they are different from that in MLV, they resemble it in that they all appear to involve a *cis*-acting sequence in the immediate vicinity of the frameshift and a large downstream structure such as a pseudoknot. It has often been proposed that this structure causes the translating ribosomes to stall, leading to the frameshift or readthrough suppression in a fraction of these events.

It is amazing to realize that while the different genera all use translational mechanisms to achieve the same result, i.e., the synthesis of a relatively large amount of Gag protein and a far smaller amount of Gag-Pol, the two mechanisms described above are completely distinct: one is an abnormal response to a termination codon, while the other occurs before the ribosomes reach the termination codon and is completely independent of the termination codon. This is but one of the myriad examples of the extraordinary diversity found among viruses.

## 5. Coda

I have here recounted four scientific stories that I was involved in; it is obvious in each case that the questions I addressed arose directly from the discoveries of the Oroszlan lab. It was an exciting time and I learned an enormous amount from my interactions with these colleagues. From a broader perspective, it is important to note that the knowledge we gained from the “basic research” on animal retroviruses, from the Oroszlan lab and many others, was of inestimable practical value: it made it possible to devise, relatively quickly, life-saving highly active antiviral therapies against the new retrovirus, HIV, when it appeared [85].

## Data Availability

Not applicable.
